# A Comparative Study of the Analgesic Efficacy of Tramadol and Diclofenac Sodium in Patients Following Cesarean Section

**DOI:** 10.7759/cureus.114633

**Published:** 2026-08-16

**Authors:** Shruti Brahmbhatt, Devashree Shah, Heena Rajput, Hitanshi B Patel

**Affiliations:** 1 Department of Pharmacology, Smt B. K. Shah Medical Institute and Research Centre, Sumandeep Vidyapeeth (Deemed to be University), Vadodara, IND; 2 Department of Obstetrics and Gynecology, Smt B. K. Shah Medical Institute and Research Centre, Sumandeep Vidyapeeth (Deemed to be University), Vadodara, IND

**Keywords:** analgesia, diclofenac sodium, post cesarean delivery, tramadol, visual analog scale

## Abstract

Introduction: Effective postoperative pain control is an essential component of patient care. Cesarean section is one of the most common surgical procedures, and adequate post-cesarean pain management is necessary to facilitate enhanced recovery, improve neonatal outcomes by promoting breastfeeding and mother-infant bonding, and reduce pain-related adverse effects. Although opioids have been the mainstay of postoperative analgesia, their adverse effects limit their use. Non-steroidal anti-inflammatory drugs have gained popularity as alternatives.

Methods: This prospective observational comparative study was conducted at a tertiary care hospital in Gujarat among female patients aged >18 years who had undergone cesarean delivery and were prescribed either tramadol or diclofenac sodium for postoperative pain management by the treating clinician. Pain was assessed using the Visual Analog Scale (VAS) at baseline and at 30 minutes, two, six, 12, and 24 hours. Adverse drug reactions were recorded. Statistical analysis was performed using standard parametric tests, with p < 0.05 considered significant.

Results: A total of 50 participants completed the study (tramadol group: n = 25; diclofenac group: n = 25), with comparable baseline characteristics. Both groups showed significant improvement in VAS scores over time (intra-group p < 0.001). Inter-group comparison demonstrated a statistically significant difference at 30 minutes, two hours, and six hours (p < 0.05), but not at 12 and 24 hours (p > 0.05). The mean reduction in VAS score at two hours was 2.4 in the tramadol group and 2.92 in the diclofenac group. The incidence of adverse drug reactions was comparable between groups (p > 0.05).

Conclusion: Both diclofenac sodium and tramadol are effective in reducing postoperative pain following a cesarean section. Diclofenac provides a faster onset of analgesia, while tramadol offers sustained pain relief. Adverse drug reaction incidence was similar in both groups, although gastrointestinal symptoms were more common with diclofenac. Analgesic choice should be individualized based on patient factors, contraindications, and clinical context.

## Introduction

Cesarean delivery is a surgical procedure that involves delivery of the fetus through incisions in the maternal abdominal wall (laparotomy) and the uterine wall (hysterotomy). The surgery is performed when vaginal birth poses a risk to the mother or baby [1].

Cesarean delivery is one of the most common surgical procedures performed worldwide and features prominently in obstetric care and has impacted positively on maternal/neonatal outcomes.

In India, the prevalence of cesarean sections had increased to 21.5% in 2021 and is expected to increase to 30% by 2030 [2]. Approximately 28%-78% of patients have reported having severe pain after cesarean delivery, which is associated with adverse outcomes like poor establishment of breastfeeding, impaired mobility, disturbed sleep, postpartum anxiety, and its consequences on maternal and neonatal well-being in the postpartum period. In rare cases, suboptimal pain management after lower segment cesarean section (LSCS) is associated with prolonged immobility and its consequences, such as deep vein thrombosis or atelectasis [3].

Pain is indicative of trauma to the tissue or disease, making effective pain relief post cesarean delivery necessary to reduce postpartum anxiety [4]. The Visual Analog Scale (VAS) is used to assess pain [5].

The administration of effective analgesia in the postoperative period is an important aspect of postoperative care, ensuring patient comfort and facilitating early mobilization and recovery. In obstetrics, the most common surgery is LSCS, for which the choice of analgesic is important due to its potential impact on both the mother and the neonate.

In the post-operative period, opioids, non-opioid analgesics, and other adjuvant medications are used for pain management after LSCS under spinal anesthesia. Opioids such as tramadol, morphine, and fentanyl act on the central nervous system, though their use is often limited by concerns over respiratory depression, necessitating careful monitoring; thus, they are used mainly in severe pain. Non-opioid analgesics, including non-steroidal anti-inflammatory drugs such as diclofenac sodium and paracetamol, offer effective pain relief with a lower risk of respiratory depression. These agents act primarily by inhibiting cyclooxygenase enzymes, presenting a safer option for mild-to-moderate pain [6].

The various adverse drug reactions reported are nausea, gastrointestinal distress, drowsiness, flatulence, vomiting, epigastric distress, gastritis, headache, etc. No significant adverse reactions are observed with these drugs [7, 8].

Despite the widespread use of tramadol and diclofenac sodium for postoperative analgesia, existing literature demonstrates considerable variability in study design, dosing regimens, and outcome measures, limiting direct comparison of their efficacy. Many studies have been conducted in heterogeneous surgical populations, with relatively fewer focusing specifically on post-cesarean section patients, where effective pain control is essential for maternal and fetal well-being. Additionally, there is inconsistent reporting of onset and duration of analgesia, particularly in the early postoperative period.

Therefore, the present study was undertaken to address these gaps by providing a structured comparison of analgesic efficacy and safety between tramadol and diclofenac sodium in post-cesarean section patients with objectives as follows:

The primary objective of this study was to compare the analgesic efficacy of tramadol and diclofenac sodium in post-cesarean section patients using the VAS. The secondary objective of this study was to compare and report the incidence of adverse drug reactions with each drug.

## Materials and methods

The study was conducted and reported as per the Strengthening the Reporting of Observational Studies in Epidemiology (STROBE) guidelines for observational research reporting and to support reproducibility for reporting cohort, case-control, and cross-sectional studies [9].

The study type was comparative prospective observational in nature, conducted in the Department of Obstetrics at Smt B. K. Shah Medical Institute and Research Centre, Dhiraj Hospital, Sumandeep Vidyapeeth (deemed to be University), a tertiary care teaching hospital in Vadodara, Gujarat, India, during a fixed data collection period from 15^th^ October, 2025, to 15^th^ April, 2026. The study was initiated after approval from the Sumandeep Vidyapeeth Institutional Ethics Committee (IEC Number: EC-CT-2019-0079), on 9^th^ October, 2025, with approval number 25/42. Permission of the head of the Obstetrics and Gynecology Department and the medical superintendent was taken before the initiation of the study.

The study included patients from the obstetrics ward older than 18 years who had undergone cesarean section delivery under spinal anesthesia and were prescribed injection tramadol or injection diclofenac sodium as part of pain management post surgery by the treating physician. Patients allergic to any of the study drugs and those who refused to participate were excluded from the study. All the patients who were assessed had been clearly informed about the purpose and nature of the study in a language they could understand. They were included in the study after obtaining a written informed consent form in their vernacular language after a thorough explanation about the purpose and method of the study through a patient information sheet before enrolling them in the preoperative period. Postoperative pain was assessed using the VAS, a validated pain assessment tool. VAS, with scores ranging from 0 to 10 (where 0 stands for no pain and 10 for the worst possible pain), was explained to each patient.

All the patients underwent a routine pre-anesthetic checkup and received similar pre- and intraoperative management as per standard practice. Spinal anesthesia with 0.5% hyperbaric bupivacaine (2.2 mL) was administered using a 23G spinal needle at the L3-L4 interspace in all patients as per standard practice, thereby maintaining homogeneity across both groups. The baseline VAS score was recorded after completion of cesarean delivery and before administration of the first analgesic dose; the second reading was taken 30 minutes after drug administration, and subsequent readings at two, six, 12, and 24 hours. All the above information was recorded along with demographic details in case record form. No patient needed rescue analgesia during the 24-hour postoperative observation period.

Sample size calculation

A prospective consecutive sampling method was applied to minimize selection bias. Every patient undergoing a cesarean section under spinal anesthesia during the study period was sequentially evaluated. A total of 84 patients were initially assessed for eligibility. Out of these, 34 patients were excluded based on predefined criteria, such as 17 who did not meet the inclusion criteria, 10 who refused to participate, and seven who were dropped due to incomplete data.

**Figure 1:**
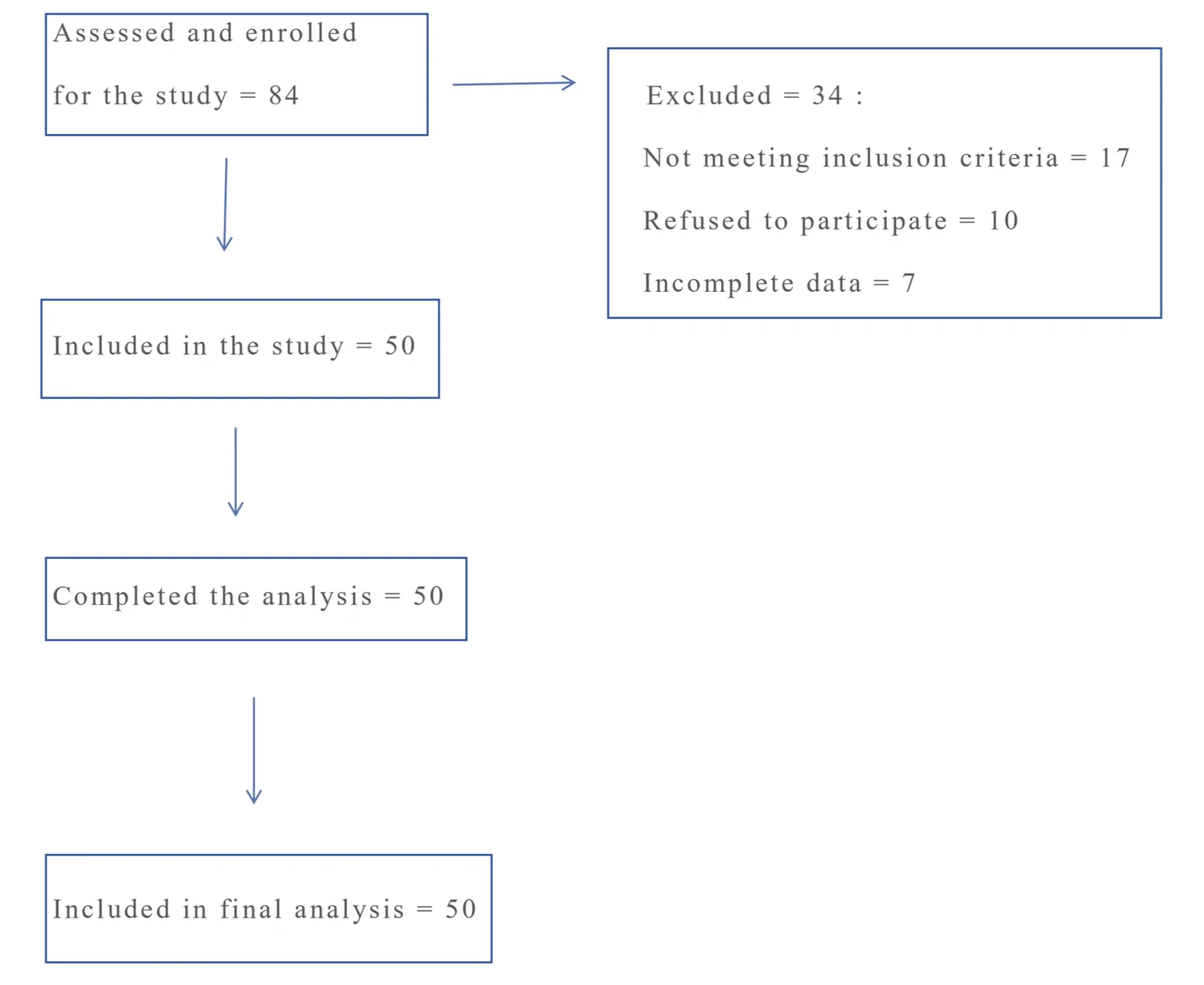
A flowchart outlining the patient selection process Out of a total of 84 assessed patients, 50 completed the study and were enrolled in group A (tramadol group) & group B (diclofenac sodium group) based on treatment prescribed by the physician.

Consequently, 50 patients successfully completed the study and were included in the final analysis. They were enrolled in Group A (tramadol group) and Group B (diclofenac sodium group) based on treatment prescribed by the physician; group allocation was completely non-interventional and determined solely by the independent prescribing decisions of the treating physicians. The flowchart for the following is presented in Figure 1.

Group A consisted of 25 patients who received 100 mg of intravenous tramadol every eight hours. Group B consisted of 25 patients who received 75 mg of intravenous diclofenac sodium every eight hours.

Statistical analysis was performed using Microsoft Excel 2021 (Microsoft Corporation, Redmond, WA, USA). Descriptive statistics were calculated, and inferential statistical analysis was conducted using the unpaired t-test and one-way analysis of variance (ANOVA) wherever applicable. A p-value of <0.05 was considered statistically significant. Data were tested for normality; upon confirmation, appropriate parametric tests were performed. Both descriptive statistics and appropriate inferential statistical tests were used to assess baseline comparability between the study groups. Continuous variables were summarized as mean ± SD and categorical variables as frequency and percentage. Inter-group comparisons were performed using an independent-samples t-test for continuous variables and a chi-square test for categorical variables, with longitudinal within-group comparisons performed using repeated-measures ANOVA. A p-value of <0.05 was considered statistically significant.

## Results

A total of 50 patients completed the study, with Group A (n=25) receiving tramadol and Group B (n=25) receiving diclofenac sodium.

The mean age was 24.52+3.11 years in Group A and 25.01+4.50 years in Group B (p=0.66). The mean weight was 55.36±3.73 in Group A and 55.84±4.21 kg in Group B (p=0.67). The mean height was 153.4±4.64 cm in Group A and 154.92±3.89 cm in Group B (p=0.21). 

Baseline VAS scores were comparable, with 7.68+0.94 in Group A and 7.32+0.94 in Group B (Table 1).

**Table 1:** Baseline characteristics of the study group

Variables	Group A (tramadol; n=25)	Group B (diclofenac; n=25)	p-value
Age (years)	24.52+3.11	25.01+4.5	0.66
Weight (kg)	55.36+3.73	55.84+4.21	0.67
Height (cm)	153.4+4.64	154.92+3.89	0.21
Gravida: Primi	14 (56%)	15 (60%)	-
Gravida: Multi	11 (44%)	10 (40%)	0.77
Gestational age (weeks)	38.81+0.73	38.51+0.98	0.25
Geographical distribution: Rural	17 (68%)	15 (60%)	-
Geographical distribution: Urban	8 (32%)	10 (40%)	0.55
Baseline Visual Analog Scale (VAS) score	7.68+0.94	7.32+0.94	0.19

Intra-group comparison of VAS scores over time within Group A and Group B

**Figure 2:**
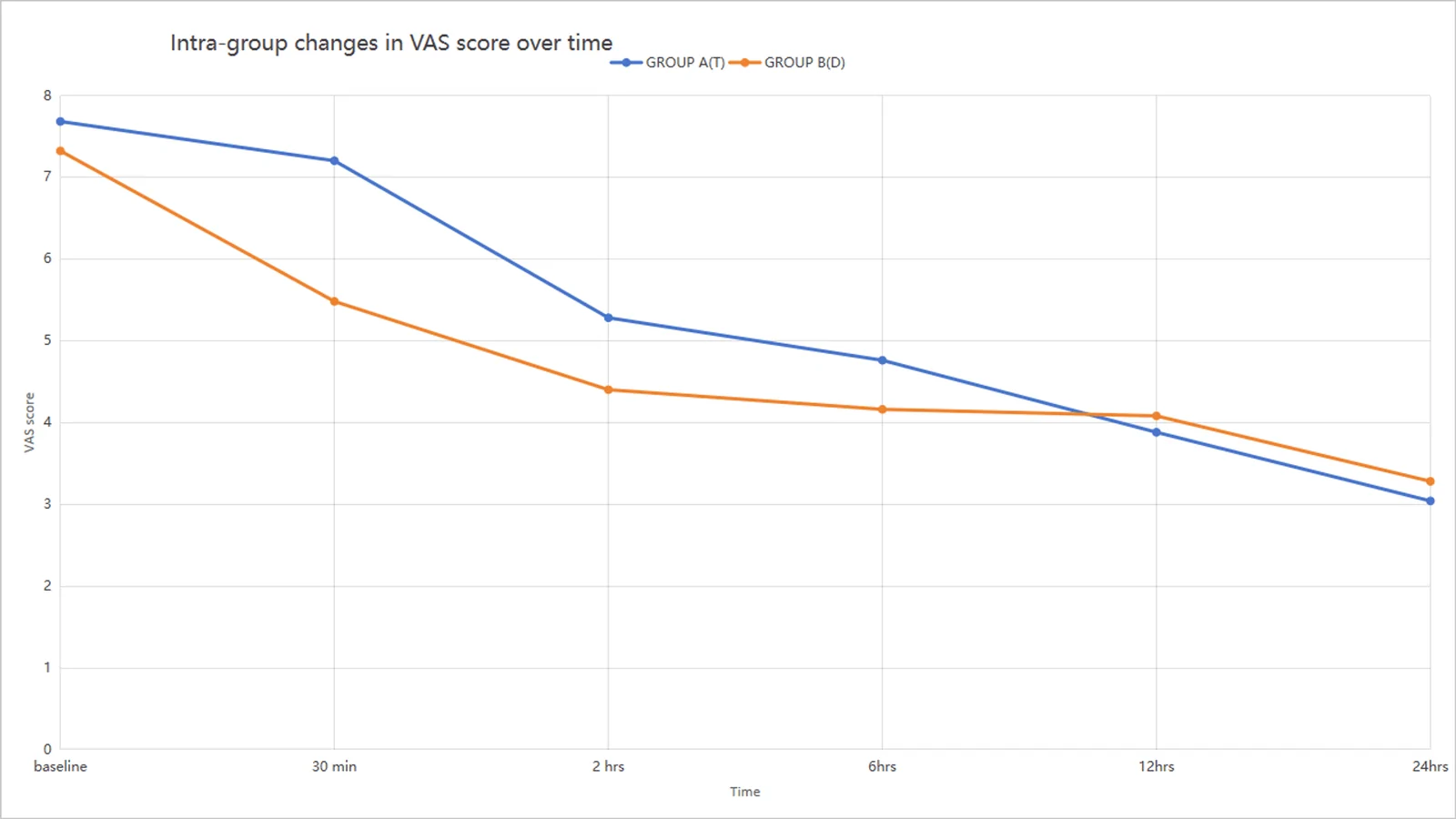
Line graph representing intra-group changes in Visual Analog Scale (VAS) score (Y axis) over time (X axis) Group A: tramadol; Group B: diclofenac

Both treatments produced statistically significant improvement in that particular group across follow-up (repeated-measures change p < 0.001 in each group) (Table 2, Figure 2).

**Table 2:** Intra-group comparison of Visual Analog Scale (VAS) scores over time within Group A and Group B Statistical test: repeated measures ANOVA (within group changes); Level of significance: p <0.05

Group	Baseline (mean)	30 minutes (mean)	2 hours (mean)	6 hours (mean)	12 hours (mean)	24 hours (mean)	p-value
Group A (tramadol)	7.68+0.94	7.2+0.81	5.28+0.61	4.76+0.59	3.88+0.43	3.04+0.53	<0.001
Group B (diclofenac)	7.32+0.94	5.48+1.12	4.4+ 1.00	4.16+0.80	4.08+0.84	3.28+0.54	<0.001

Mean reduction in VAS score from baseline to two hours was 2.4 with Group A and 2.92 with Group B.

Intergroup comparison of VAS scores between Group A and Group B demonstrated a statistically significant difference at 30 minutes, two hours, and six hours (p < 0.05). However, no statistically significant difference was observed at 12 hours and 24 hours (p > 0.05) (Table 3, Figure 2).

**Table 3:** Inter-group comparison of Visual Analog Scale (VAS) scores between Group A and Group B at different time intervals Statistical test: unpaired Student’s t-test; Level of significance: p <0.05

Time	Group A (tramadol) (Mean + SD)	Group B (diclofenac) (Mean + SD)	p-value
Baseline	7.68+0.94	7.32+0.94	0.19
30 minutes	7.2+0.81	5.48+1.12	<0.0001
2 hours	5.28+0.61	4.4+ 1.00	0.001
6 hours	4.76+0.59	4.16+0.80	0.005
12 hours	3.88+0.43	4.08+0.84	0.28
24 hours	3.04+0.53	3.28+0.54	0.12

**Figure 3:**
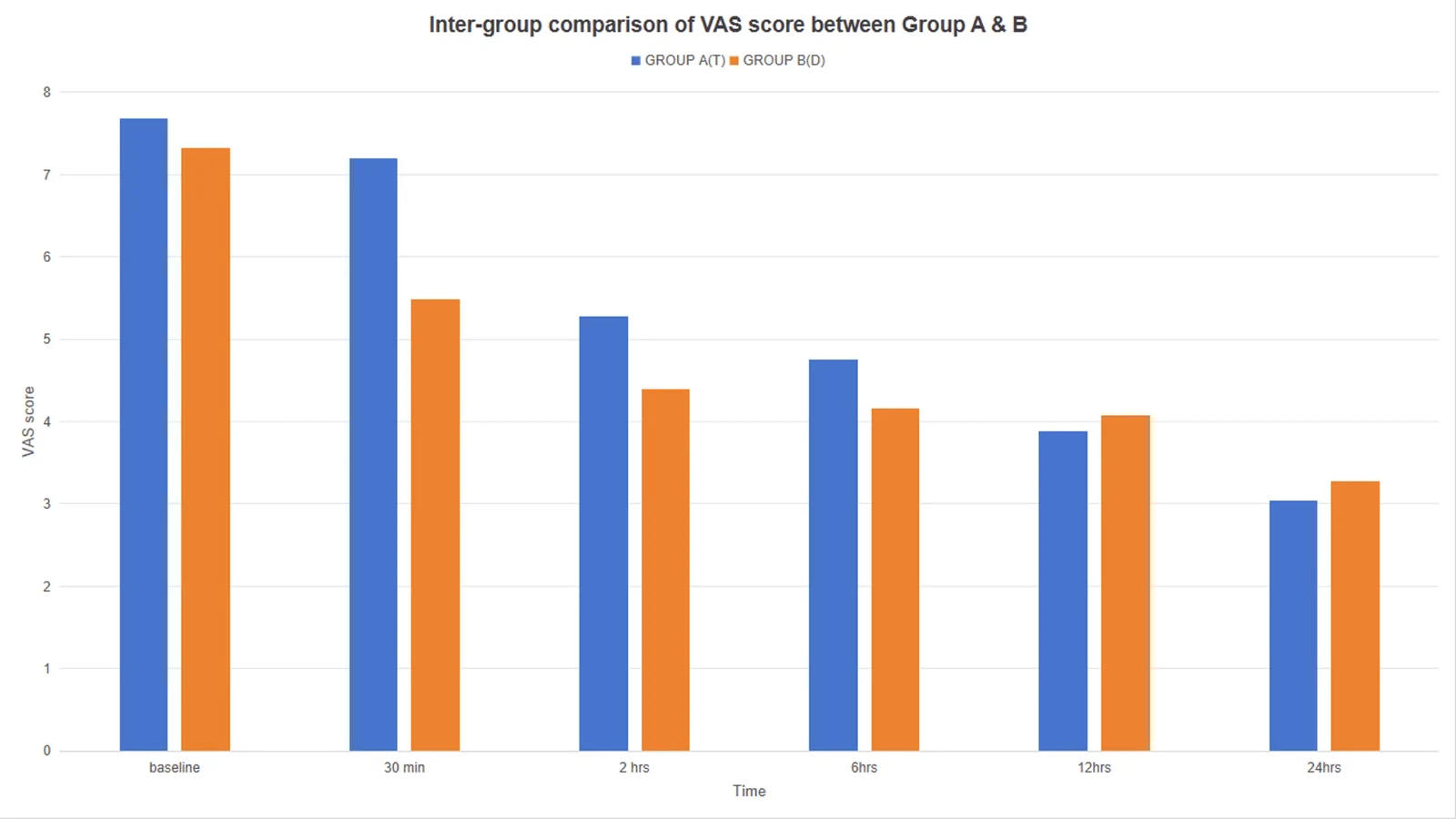
Column graph representing inter-group comparison in Visual Analog Scale (VAS) scores over time Group A: tramadol; Group B: diclofenac

Among the adverse drug reactions observed in the 25 patients per group, nausea was the most frequent in both the tramadol and diclofenac groups (six, 24% vs eight, 32%), respectively). Vomiting was reported more commonly with tramadol (four, 16% vs two, 8%), whereas gastritis was higher in the diclofenac group (six, 24% vs two, 8%) (Table 4).

**Table 4:** Incidence of adverse drug reactions (ADR) in both groups

ADR	Group A (tramadol)	Group B (diclofenac)
Vomiting	4 (16%)	2 (8%)
Gastritis	2 (8%)	6 (24%)
Nausea	6 (24%)	8 (32%)

## Discussion

Postoperative pain management following cesarean section is essential for early mobilization, improved maternal comfort, facilitation of breastfeeding, and enhanced mother-infant bonding. Recently, emphasis on the importance of effective multimodal pain control to improve maternal recovery and neonatal care has increased [10].

In the present study, both diclofenac sodium and tramadol demonstrated significant reduction in VAS scores over time, indicating that both drugs are effective analgesics in post-cesarean pain management. However, no significant differences were observed between the two groups in overall analgesic efficacy. These findings are consistent with the study conducted by Shukla et al., which reported comparable analgesic efficacy between intravenous tramadol and diclofenac sodium in postoperative pain management. Similarly, a recent randomized controlled trial by Borade et al. also demonstrated no significant difference in analgesic effectiveness between the tramadol and diclofenac sodium groups, further supporting the results of the present study [11, 12]. 

However, comparatively, diclofenac sodium showed greater analgesic efficacy at earlier time intervals, suggesting a faster onset of action compared to tramadol. This observation is consistent with the findings of Yaowalaorng et al., who demonstrated rapid analgesic efficacy of diclofenac sodium over tramadol in acute pain settings, thereby supporting the early analgesic advantage observed in our study [12, 13].

The faster onset observed with diclofenac sodium can be attributed to its favorable pharmacokinetic profile, including rapid absorption and early peak plasma concentration, leading to prompt inhibition of cyclo-oxygenase enzymes and reduction in prostaglandin-mediated pain at the site of tissue injury. In contrast, tramadol showed a relatively slower onset of action due to its central mechanism and requirement for metabolic activation to its active metabolite. It acts through μ-opioid receptor agonism along with inhibition of serotonin and norepinephrine reuptake, resulting in a delayed onset but sustained analgesic effect. Thus, while diclofenac sodium is effective in controlling early postoperative pain, tramadol provides prolonged analgesia through centrally mediated mechanisms [12, 14].

These pharmacological differences explain the faster onset of diclofenac sodium and the sustained analgesic effect of tramadol.

In the present study, the incidence of adverse drug reactions was comparable between the two groups, with no significant difference observed. However, gastrointestinal adverse effects were more commonly reported with diclofenac sodium. These findings are consistent with Kumar et al., who reported a higher incidence of gastrointestinal side effects with diclofenac sodium compared to tramadol. Similarly, Borade et al. observed that both drugs were generally well tolerated, with comparable safety profiles, further supporting the results of the present study [12, 15].

The strengths of this study include adherence to STROBE guidelines and direct comparison of two commonly used analgesics, diclofenac sodium and tramadol, in a clinical setting, making the findings relevant to routine practice. The study addresses a clinically relevant issue, as effective postoperative pain management after a cesarean section plays a crucial role in improving maternal outcomes, including early breastfeeding and recovery. A consecutive sampling method was used to reduce selection bias. Pain assessment was performed using a standardized and widely accepted tool, the VAS, at multiple time intervals, allowing for consistent evaluation of analgesic efficacy. The comparable baseline characteristics between our study groups support reasonable demographic comparability for our analytical cohorts, and no rescue analgesia was needed by any patients; thus, it does not affect confounding.

The study also included an assessment of both analgesic efficacy with monitoring and reporting of adverse drug reactions, providing a more comprehensive evaluation of the drugs. Tramadol is associated with a range of psychological symptoms, including manic episodes, serotonin syndrome, psychosis, and cognitive impairment. Risk factors included age, pre-existing psychiatric conditions, polydrug use, and prolonged tramadol use, especially in vulnerable populations. Clinicians should closely monitor patients for these adverse effects [16]. Diclofenac has a complex adverse drug profile, causing gastrointestinal system issues, rashes, edema, etc. The most damaging were peptic ulcers with or without perforation and, in rare cases, acute renal failure; Stevens-Johnson syndrome was even reported. So the study shows the importance of pharmacovigilance even on most prescribed medicines [17].

Limitations

This study had limitations, such as the sample size being relatively small. The observational study design without randomization or blinding introduces the possibility of residual confounding variables like individual baseline pain thresholds, psychological anxiety, etc.

Additionally, the duration of follow-up was limited to 24 hours postoperatively, restricting the assessment of long-term analgesic efficacy and delayed adverse drug reactions. 

This study was conducted entirely at a single tertiary care teaching hospital in Gujarat, India, which may restrict the generalizability of the findings to other healthcare settings.

## Conclusions

In this study, both tramadol and diclofenac sodium demonstrated a significant reduction in VAS scores over time, indicating effective pain relief in both groups. However, diclofenac sodium exhibited a faster onset of analgesic action and showed a significant clinical association with early pain relief in controlling early postoperative pain. In contrast, tramadol provided centrally mediated analgesia with a sustained effect. Both drugs were generally well tolerated, with mild adverse effects. Gastrointestinal symptoms were more commonly observed with diclofenac sodium. 
